# “Well advised” – Simulation of an ethical case consultation with students of evidence-based nursing, midwifery, and human medicine as part of an interprofessional education course

**DOI:** 10.3205/zma001825

**Published:** 2026-03-23

**Authors:** Christiane Vogel, Jan Schildmann, Sabine Sommerlatte, Elisabeth Schmidt

**Affiliations:** 1 Martin Luther University Halle-Wittenberg, Medical Faculty, Institute for History and Ethics of Medicine, Halle (Saale), Germany; 2 Martin Luther University Halle-Wittenberg, Medical Faculty, Institute for Rehabilitation Science, Halle (Saale), Germany

**Keywords:** interprofessional education (IPE), principle-based ethics case consultation, professional identity formation, interprofessional collaboration (IPC), collaborative practice

## Abstract

**Aim:**

Interprofessional education (IPE) prepares students in health professions for the joint care of patients and promotes (inter)professional identity formation. Interprofessional collaboration in clinical-ethical case consultations is essential for the best possible recommendations for action. Therefore, simulating the already established format of principle-oriented ethical case consultation is ideal for IPE.

**Method:**

An interprofessional education course was developed for three professions (evidence-based nursing, midwifery, and human medicine) with a focus on simulating principle-oriented ethical case consultations. After a theoretical introduction, students act in their (future) professional roles as participants in such a consultation. Two cases are simulated and reflected upon using a modified fishbowl method. The course is evaluated using a self-developed questionnaire implemented with EvaSys.

**Results:**

The course has been held three times since the winter term of 2022/2023. Ninety-six evaluation forms were evaluated (78% response rate) from 123 students. Students reported, *inter alia*, a “positive change in attitude toward interprofessional collaboration through the course” with an average of 1.8±0.8 and an “awareness of various ethical dimensions of professional activity” with 1.6±0.9 on a 5-point Likert scale (1=“completely agree”). The free-text responses show that the students particularly benefited from the practical exercises, the real-life cases, and the interprofessional exchange.

**Conclusion:**

Ethical issues are a natural focus for IPE due to the multi-professional perspective they require. This allows for the promotion of practical understanding and appreciation of various ethically relevant perspectives.

## Zusammenfassung

**Zielsetzung:**

Interprofessionelle Lehre (IPL) bereitet Studierende der Gesundheitsberufe auf die gemeinsame Versorgung von Patient:innen vor und fördert die (inter-)professionelle Identitätsentwicklung. Für bestmögliche Handlungsempfehlungen ist eine interdisziplinäre Zusammenarbeit in klinisch-ethischen Fallberatungen unerlässlich. Daher bietet sich die Simulation des bereits etablierten Formats der prinzipienorientierten ethischen Fallberatung für die IPL an.

**Methodik:**

Es wurde eine interprofessionelle Lehrveranstaltung (LV) für drei Professionen (Evidenzbasierte Pflege, Hebammenwissenschaft und Humanmedizin) entwickelt mit Fokus auf der Simulation von prinzipienorientierten ethischen Fallberatungen. Nach einer theoretischen Einführung agieren die Studierenden in ihren (zukünftigen) beruflichen Rollen als Teilnehmende einer solchen Beratung. Mittels einer modifizierten Fishbowl-Methode werden zwei Fälle simuliert und reflektiert. Die LV wird mit einem selbstentwickelten Fragebogen evaluiert, der mit EvaSys umgesetzt wird.

**Ergebnisse:**

Die LV wurde seit Wintersemester 2022/2023 drei Mal durchgeführt. Von 123 Studierenden konnten 96 Evaluationsbögen ausgewertet werden (Rücklauf 78%). Auf einer 5-stufigen Likertskala (1=„trifft völlig zu“) berichten die Studierenden u.a. über eine „positive Veränderung der Haltung zur interprofessionellen Zusammenarbeit durch die LV“ mit im Mittel 1,8±0,8 und die „Sensibilisierung für verschiedene ethische Dimensionen für die berufliche Tätigkeit“ mit 1,6±0,9. Die Freitexte zeigen, dass die Studierenden besonders von der praktischen Übung, den echten Fällen und dem interprofessionellen Austausch profitiert haben.

**Schlussfolgerung:**

Ethische Themen bieten sich aufgrund der erforderlichen multiprofessionellen Perspektive als inhaltlicher Fokus für die IPL an. Hier kann praxisnah Verständnis und Wertschätzung verschiedener ethisch relevanter Perspektiven gefördert werden.

## Introduction

Well-functioning interprofessional collaboration (IPC) between members of different healthcare professions has improved patient care and made it safer [1], [2], [3], [4]. It is important for practical implementation that interprofessional education (IPE), i.e., learning about, from, and with each other with two or more professions, is already incorporated into studies and training [5]. Participants become more familiar with each other’s communication and thinking styles through joint interaction between professional groups. In addition to their personal and professional skills, students also broaden their horizons regarding the “integration of knowledge, skills, and values that define interprofessional collaboration”; for example, relationship-, process-, and results- orientation, as well as mutual respect and teamwork [6]. This form of collaboration can, thus, be seen as a driving force for personal identity formation, particularly in the early stages of training. In addition to a positive influence on professionalism, well-being, and inclusivity, Sternszus et al. (2023) see the development of a greater value awareness and meaning within IPC [7]. Interprofessional identity refers to the sense of belonging to an interprofessional community, which can be fostered particularly in spontaneous, authentic clinical settings [8]. The IPE needs to be incorporated early on into the training of healthcare professionals for a long lasting impact on interprofessional identity. 

Interprofessional collaboration ranges from the exchange of ideas and knowledge to mutual support and joint task processing, and implies varying degrees of intensity and mutual dependencies [9]. One example of an intensive and multifaceted form of interprofessional cooperation in the healthcare sector is clinical ethics case consultation – a cross-professional care concept that requires strong networking. Ethics case consultation is understood as “support for all persons involved in healthcare in ethically difficult or challenging situations [and] deals primarily with issues relating to the care of individuals” [10]. This is where various types of knowledge in terms of professional expertise meet individual values shaped by one’s profession. An interprofessional teaching unit (TU) focusing on the simulation of such ethical case consultations can raise the awareness of this form of cooperation at an early stage. The focus is on working together, solving problems, discussing and reflecting with each other, and getting to know other perspectives and experiences. At the same time, important soft skills are taught, such as mindfulness, curiosity, and stress tolerance, which are needed in the context of ethical case consultation.

Students in healthcare professions are confronted with ethical challenges both during their studies and later in their careers [11]. Challenges arise, *inter alia*, from the “growing demands and increasingly complex needs of patients” that transcend professional boundaries [12]. In recent years, many healthcare facilities have established structures for providing advice on ethical conflicts [13]. An important prerequisite for the professional implementation of ethical case consultation is the participation of ethically competent representatives from various healthcare professions, who consult together and contribute their different (moral) perspectives and experiences. This is necessary to ensure that medical and nursing aspects of the situation are addressed at the beginning of each case consultation, because “good ethical decisions can only be made on the basis of morally relevant nursing and medical facts” [14].

**Figure 1:**
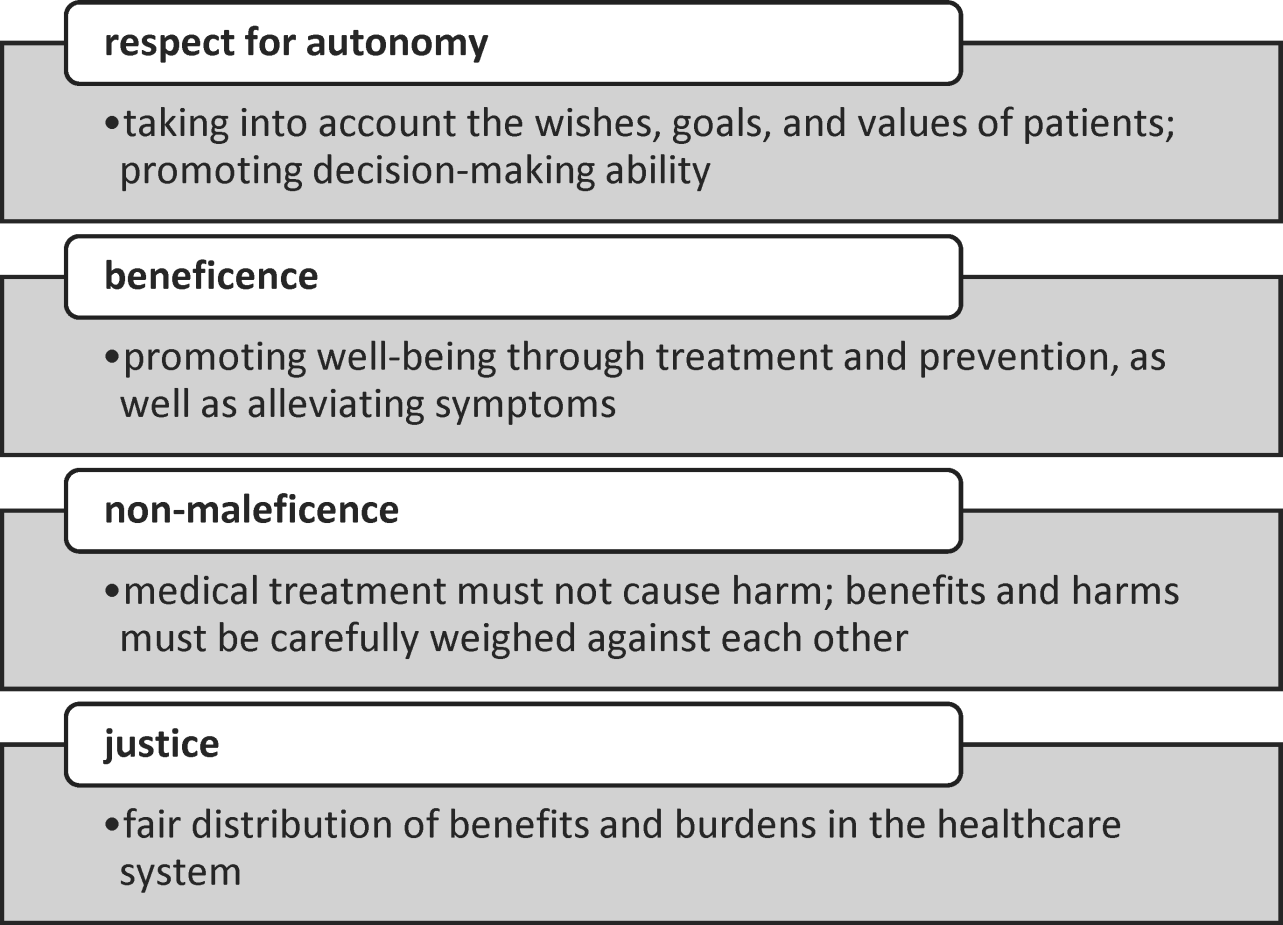
Four mid-level principles according to Beauchamp and Childress [17], including supplementary descriptions according to [16]

A structured method for dealing with an ethical conflict is a principle-based case consultation [15], [16], which is based on the four fundamental principles of ethical conduct in medicine (see figure 1 (Fig. 1)) developed by Beauchamp and Childress [17].

These principles provide a framework for approaching a specific ethical problem [14]. 

Participation in an ethical case consultation may reveal potential discrepancies between aspects of personal identity and certain norms of one’s own and other professions. Participation can help students to develop an increasingly complex understanding of themselves as individuals and in their role as healthcare professionals with an authentic and open-minded self-image [7]. The reflection process begins particularly in nonroutine situations, such as in an ethical case consultation, so that “in the best case, this leads to a critical analysis of one’s own knowledge and self-perception” [18].

Simulating a structured, principle-based ethical case consultation provides a safe learning environment to support students in developing their (inter)professional identity and teaching them skills for situations where they need to take responsibility (see table 1 (Tab. 1)) and dealing with challenging situations in an interprofessional setting [19]. 

## Aim

Our aim is to design, implement, and evaluate an IPE event that raises the awareness of ethical issues and IPC among students in evidence-based nursing (EBN), midwifery, and human medicine (HM) programs. The focus is on simulating principle-oriented case consultations and teaching (inter)professional skills. The findings will be used in evidence-based teaching for the future design and implementation of IPE on ethical issues in healthcare. The National Competence-Based Learning Objectives (NKLM) Catalogue 2.0 serves as a reference point for the development of future teaching and learning content.

## Project description

### Located at the Martin Luther University Halle-Wittenberg 

Interprofessional education is a chosen focus at the Medical Faculty at the Martin Luther University Halle-Wittenberg [20]. Various interprofessional modules are currently established in teaching, particularly in the HM and EBN degree programs and the nursing and physiotherapy apprenticeships [20], [21]. Other professional groups are to be included in the IPE, especially students of midwifery. The focus of IPE in the HM degree program is within the practical year: an IPE module of 240 minutes must be taken during each trimester. Practical year students can choose from the modules offered. Various IPE modules are incorporated into the curriculum on a longitudinal basis in the EBN degree program, starting in the third semester. The new midwifery degree program, established in 2021, is currently being integrated into the IPE.

The ethics and history of medicine module is part of the EBN and midwifery degree programs in the third semester and taught jointly. It made sense to establish an IPE event as part of this module. Human medicine students were included as the third professional group. For organizational reasons, these students are in their eleventh semester, within their practical year.

### Planning the course 

The IPE course was designed and conducted by an interprofessional team: CV (philologist, ethics consultant, teaching experience), JS (physician, medical ethicist, ethics consultant, teaching experience), SS (physician, ethics consultant, teaching experience), ES (IPE teaching coordinator, health scientist, physiotherapist, IPE subject expert, teaching experience). Thanks to our excellent cooperation with neighboring institutes, we are always able to draw on specific expertise from the relevant professional fields. 

As has already been described, there were various challenges throughout the phase of planning the course:

- compatibility of different curricula
- scheduling (including the planning team)
- organizational effort
- elective subject HM located within the summer term, ethics for midwives and EBN students within the winter term
- suitable case vignettes

### Course structure and implementation 

**Figure 2:**
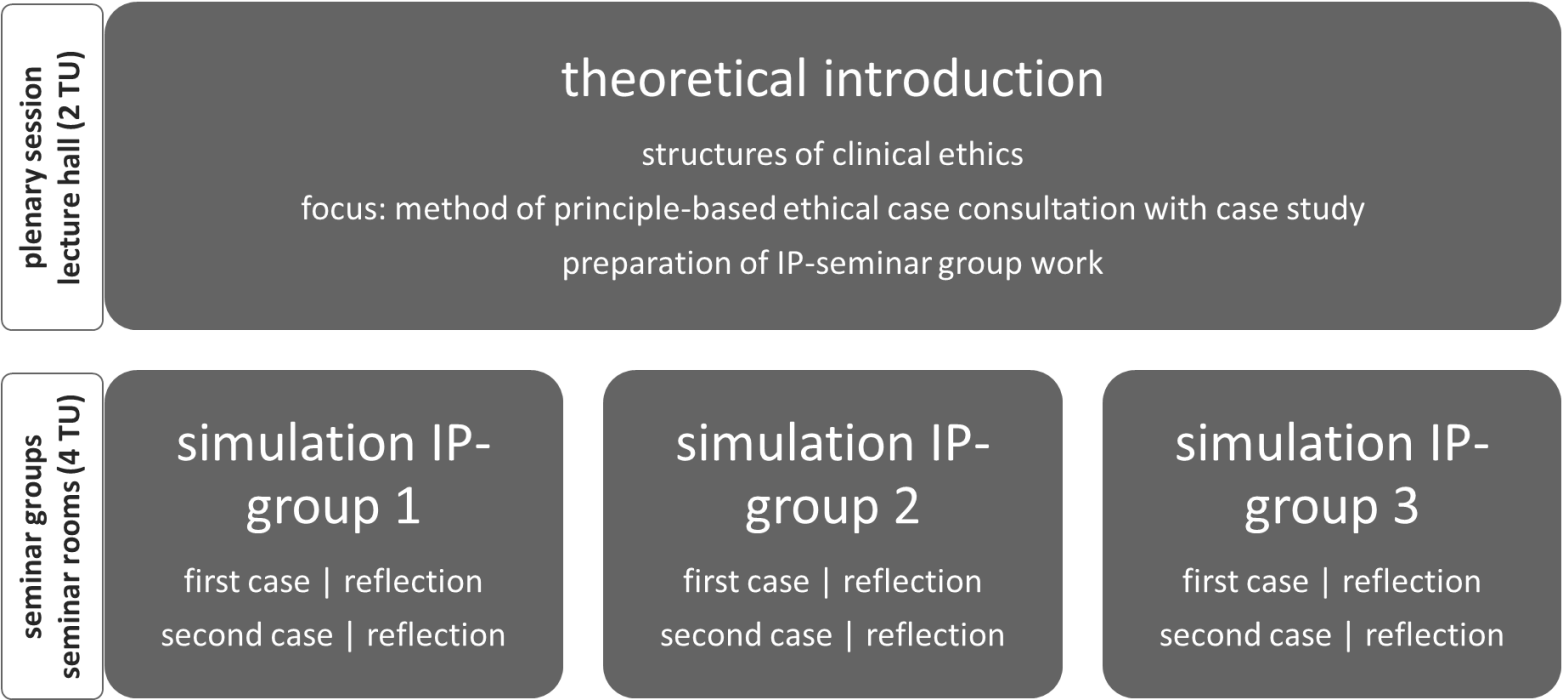
Overview of the course structure (own illustration)

A typical feature of IPE is that it is often “supervised by several teachers [...] in person and [...] characterized by predominantly interactive teaching formats or a combination with frontal teaching” [22]. A combination of frontal theoretical introduction and interactive teaching format has proven to be beneficial in our course (see figure 2 (Fig. 2)).

The course started with an introduction to clinical ethics and an overview of the subject catalog of the clinical ethics committee at the University Medicine Halle in order to familiarize students with the services offered there. This was followed by a theoretical introduction to principle-based ethics (see above). The method of principle-based ethical case consultation was applied jointly using a case study that focused on weighing the benefits and harms of continuing treatment for a patient in intensive care. In addition, the lecturer encouraged an exchange on the expectations and challenges of interprofessional case consultation by asking questions about possible initial personal experiences with ethical case consultation, for example, during an internship. If there were no personal experiences, students were asked to discuss the challenges and their own expectations regarding participation in an interprofessional ethical case consultation. This took approximately 10-15 minutes.

With an equal distribution of professions, the group moved from the plenary session into three seminar groups. Here, two anonymized and modified cases from the University Medicine Halle’s clinical ethics committee ethics advisory service were simulated by the students working together in their respective (future) professional roles. The first case focuses on the ethical dilemma involving a patient who refuses a life-saving blood transfusion on religious grounds. The second case discusses an ethical conflict in neonatology. The focus is on jointly determining the best interests of the child, weighing up the benefits and harms of continuing intensive care measures in a case of a borderline indication. Students take on both the role of participants in an ethical case consultation and that of moderators.

**Figure 3:**
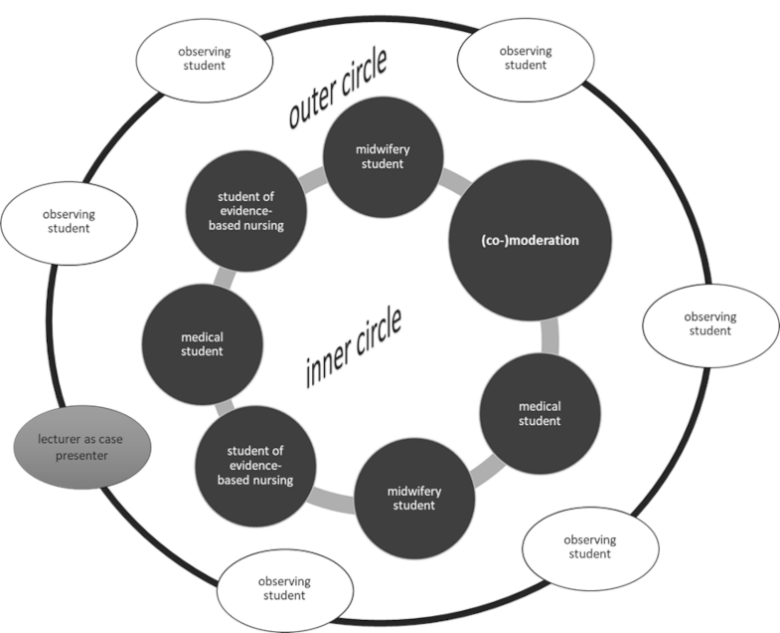
Modified version of the fishbowl method (own illustration)

A slightly modified version of the fishbowl method is used for this purpose, with (co-)moderation and active participants in the “inner circle” who can present and contribute their professional perspectives on an equal footing, as well as passive participants (observers) in the “outer circle” who focus on providing feedback (see figure 3 (Fig. 3)).

Moderation and counseling were reflected upon by the course instructors and students in a semi-structured group discussion after each “case” had been completed. The groups had approximately one TU available for this out of four. Observation and reflection questions covered the role of the moderator (“What went well?,” “What did you feel was missing?,” “What should be considered next time?”) or ethical reasoning based on the specified consultation structure. Students were given a handout to assist them containing the most important points on principle-oriented ethical case consultation and moderation. After the lecture, evaluation forms were distributed, filled out voluntarily and anonymously, and collected again.

### Evaluation

The course was evaluated using a self-developed questionnaire with open and closed questions, which was implemented with EvaSys. The questions relating to IPE are based on the specified core competencies of IPEC [6]. The primary focus is on collecting the subjective experiences and perceptions of the participants. The domains of the questionnaire include personal details, the learning effect, organization and structure of the course, learning content/prior knowledge, and overall assessment. The latter domain also includes open-ended questions. Closed questions were answered using a Likert scale from 1 to 5. Mean values and standard deviations, *inter alia,* were calculated. 

## Results

The IPE event “ethical case consultation” was held in the winter terms of 2022/2023, 2023/2024, and 2024/2025. 

Ninety-six evaluation forms were evaluated (n=96, response rate 78%) out of a total of 123 students. All forms, including those that were incomplete, were included in the evaluation. Selected results are presented in this paper.

**Figure 4:**
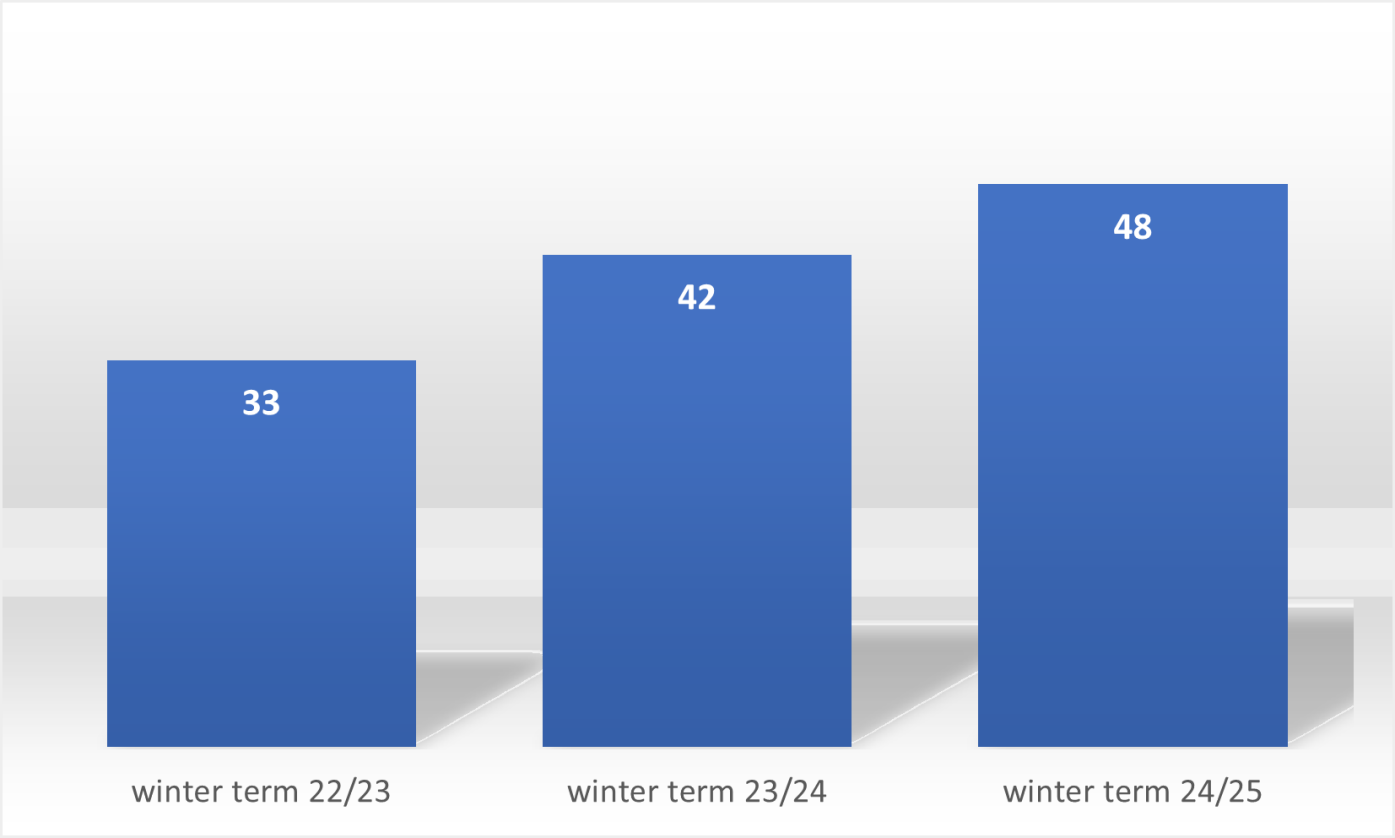
Total number of students over the course of the winter terms

**Figure 5:**
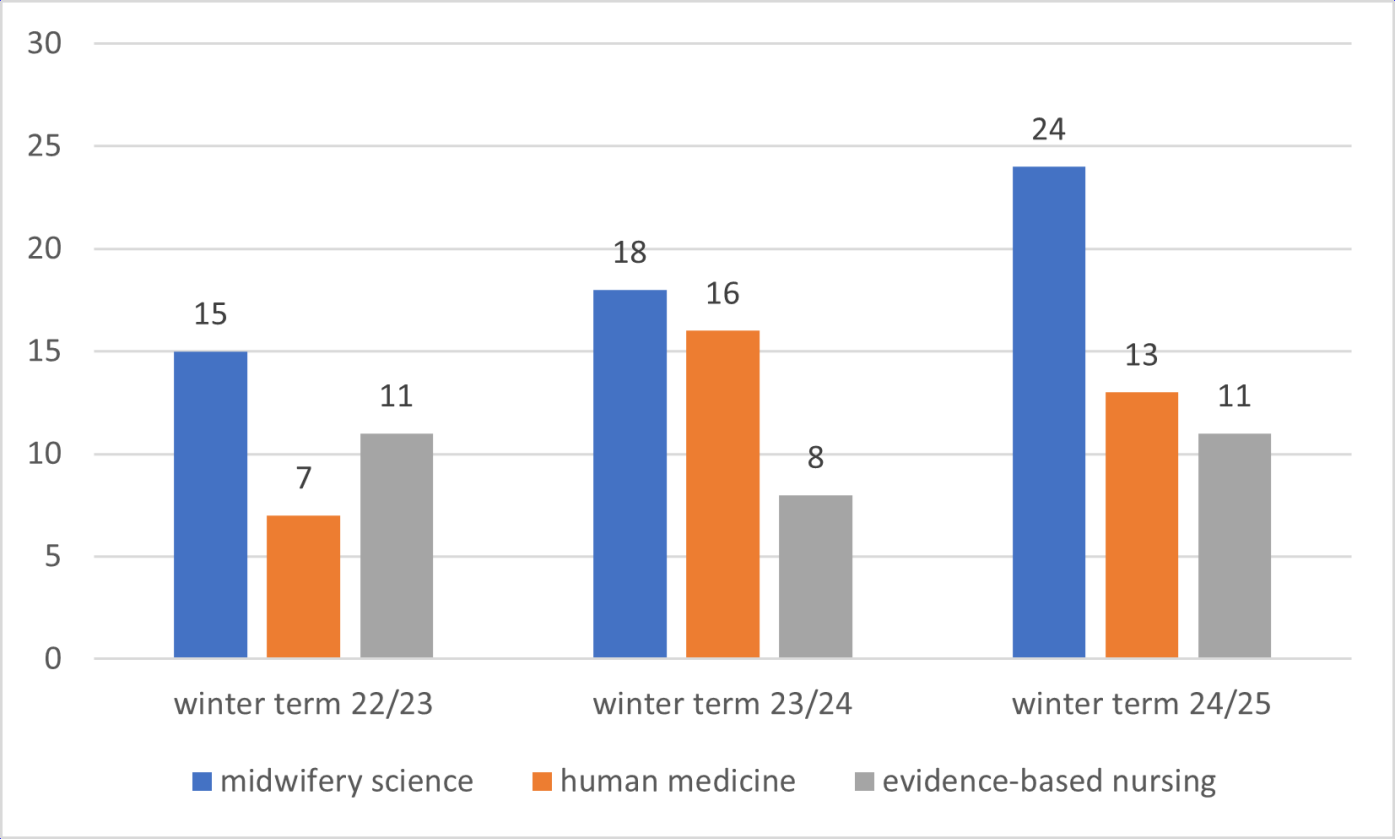
Overview of the numerical distribution of fields of study and training over the course of the winter terms

Figure 4 (Fig. 4) and figure 5 (Fig. 5) provide an overview of the participating students across the various terms and the numerical distribution of fields of study and training.

With a total of 96 evaluations, students of midwifery science predominate (46%, n=44), followed by those of HM (31%, n=30) and of EBN (23%, n=22). 

“The didactic structure was appealing” was rated at 2.1±0.9 on a 5-point Likert scale (1: “completely agree”), “organization of the module sequence” at 1.8±1, and “appropriate group size” at 1.7±0.9. The “content amount” was rated at 2.9±0.6 (Likert scale 1–5 from “too large” to “too small”). 

**Figure 6:**
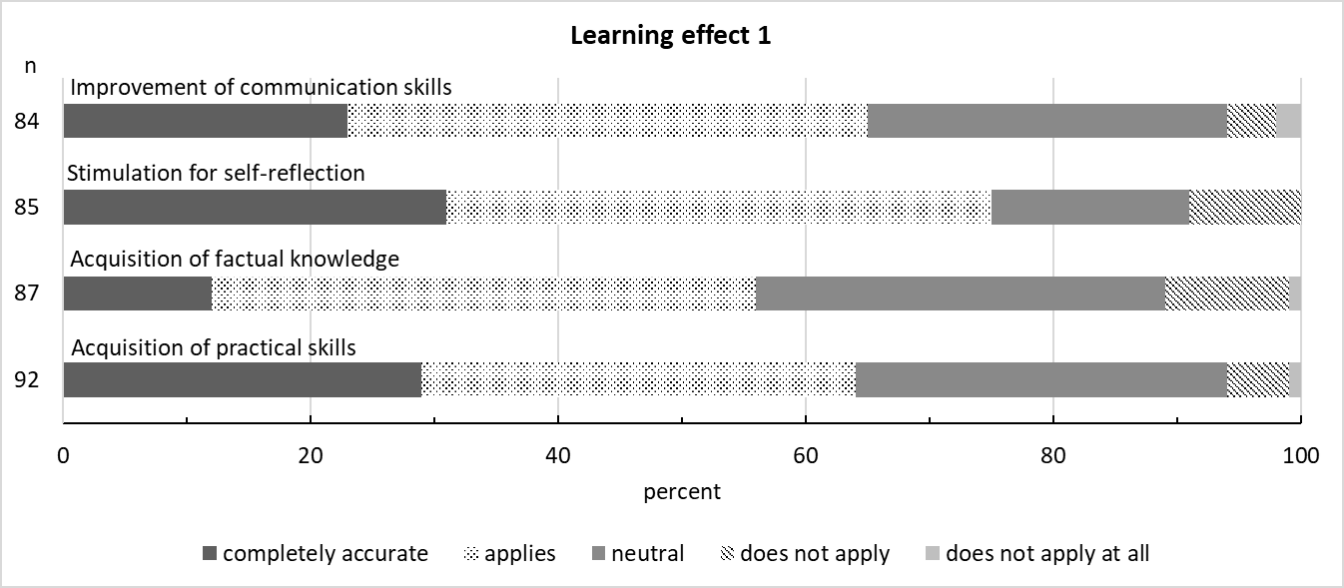
Learning effect 1 (n is the number of answers given)

**Figure 7:**
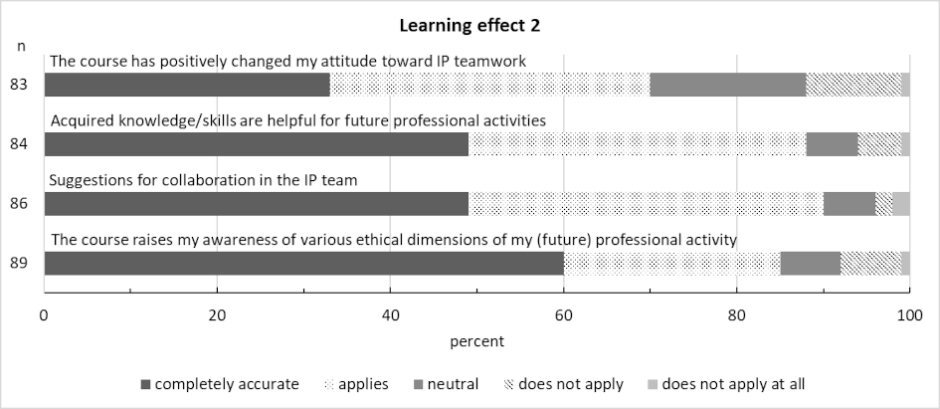
Learning effect 2 (n is the number of answers given)

The results for the domain of learning outcomes are shown in figure 6 (Fig. 6) and figure 7 (Fig. 7).

Responses to the open-ended questions (“What did you particularly like?,” “What would you have liked to be different?”) were summarized in terms of content. The most important findings are presented in table 2 (Tab. 2).

Regarding the overall assessment of the evaluation, 100% (n=80) of students would like this course to continue, and 96% (n=76) would like further subject-specific IPE courses to be offered. Overall, they rate the course with an average of 1.7±0.5 (n=75) on a school grading scale of 1-5 (1 being the best grade).

## Discussion

The IPE event on clinical ethics case consultation has been evaluated very positively so far. When interpreting the data, it should be noted that the number of cases is limited. Due to the nature of the evaluation, with the exception of sociodemographic data, the results can only be presented collectively for all professions. This should be considered in a differentiated manner in the future.

Evaluation responses show that students report improvement in three of the four main IPEC categories (values and ethics, communication, teams and teamwork) [6]. The course, thus, contributes to an increase in competence in these areas. The fourth category (roles and responsibilities) is not explicitly addressed in the evaluation. It can be assumed that the intensive exchange between students and the respective subject areas and expert opinions also lead to a broader understanding of roles and responsibilities. A question on this topic could be added to future evaluations.

As expected, items relating to the acquisition of factual knowledge and practical skills were rated in the middle range, as the course did not focus on improving these areas. The learning effect for the items relating to interprofessional and ethical dimensions was rated and assessed significantly better. This shows that the course achieved its objectives in this regard. Almost all students would like to see this and other IPE courses offered. It can, thus, be assumed that IPE and IPC are important to students both for patient care and their own professional development.

The high proportion of midwifery students is due to organizational reasons and the size of the respective year group. The authors have no influence on this. The composition of the student body, therefore, varies from year to year. Both cases planned for the simulation have relevant points of reference to ensure that students of midwifery science feel well integrated. 

The joint theory section ensures that all students have the same level of knowledge on the topic. This is an attempt to even out the differences in educational backgrounds. Furthermore, students of both midwifery science and EBN have already been able to gain practical experience as part of their curricula. It would be desirable for students from different degree programs to have a similar level of training, for example, for prospective medical students to be able to participate in the course in an earlier semester. This is not possible at the present time for organizational reasons.

Ethical issues are ideal topics for IPE due to the multi-professional perspective they require. The results presented here underscore the added value that simulating ethical case consultations brings to students from different professions – also regarding personal and professional development (professional identity formation). This means that the ethical and communication skills acquired in lectures on ethical case consultations can be used both during studies and later in the profession. Our results are in line with existing publications on IPE with an ethical focus [23], [24].

Sternszus et al. (2023) also emphasize the positive influence of IPC on professionalism, well-being, and inclusivity [7]. Seidlein and Salloch (2025) recently highlighted the “good opportunities to promote knowledge and understanding in both professional groups” if interprofessional teaching formats are “not limited to practical topics of diagnosis and treatment” [25]. The competencies and learning objectives implemented in our course can also be found in the National Competence-Based Learning Objectives Catalogue 2.0 [https://nklm.de/zend/objective/list/orderBy/@objectivePosition/modul/200553], both in part VIII.6, Professional conduct and ethics, history and law of medicine and in part VIII.3, Interprofessional competencies. In particular, the action competencies and IP competencies NKLM VIII.3-01.2 (value-oriented interaction within the IP team) and NKLM VIII.3-03 (team communication) were strengthened through the independent implementation of ethics case consultation (NKLM VIII.6-04.4.12) and reflection on it. The depth of competence is assessed as 3b, and as 3a to 3b in the areas of ethics. The requirements of the NKLM are at least met or exceeded. It can be assumed that competence development is equally relevant for midwifery science and EBN and takes place in a similar manner.

### In- and output of individual professions – findings

In order to improve the quality of care and patient safety, IPE modules should be integrated into the training of healthcare professions at an early stage. The shared nature of these important overarching goals can be created through a “culture of cooperative interdependence and collaboration between teams” [26]. Interprofessional learning is essential for the development of one’s own professional identity. This also applies to a better understanding of the roles and identities of others. According to Juschka et al. (2024) [22], “no profound understanding of the different roles and cultures of the other professions” can be developed without intensive cooperation between the professions. An example of this is the input from a prospective midwife who mentioned that in the event of discontinuation of therapy in neonatology, an emergency baptism or taking a footprint as a possible memento should be considered, especially regarding the social environment and the parents. These are important aspects that should be taken into account by the treatment team in such a situation.

Reflecting after the simulation enables an awareness of the process, which is where the actual learning takes place. Reflection can relate to the case discussed, to one’s own (future) professional position, and to one’s role within the team. Realizing one’s own point of view and representing it authentically, as well as becoming aware of other positions and questioning them critically, is also part of reflection [6].

Regarding the evaluation, there are considerations to adapt the event for the future. The possible adaptation relates particularly to the theoretical part, which could, for example, be preceded by blended learning or a flipped classroom. This would allow sufficient space for students to acquire new knowledge at their own pace and also consider their different levels of prior knowledge. Another adaptation relates to the requirements for the case design of the specifically prepared vignettes in order to do justice to all professions and ensure that no one is lost or excluded in the simulated counseling process. Furthermore, fact sheets on the respective cases can be made available to students.

## Conclusion

Interprofessional education for ethical case consultation promotes the understanding and appreciation of different ethically relevant perspectives and ethical assessments of actors in the healthcare sector. Ethical topics are ideal as a content focus for IPE due to the necessary multi-professional perspective. Our event is continuously evaluated. The results obtained are used in the spirit of evidence-based teaching for the future design and implementation of IPE events on ethical issues in healthcare. 

## Authors’ ORCIDs

- Jan Schildmann: [0000-0002-5755-7630]
- Elisabeth Schmidt: [0009-0006-2087-0371]

## Competing interests

The authors declare that they have no competing interests. 

## Figures and Tables

**Table 1:**
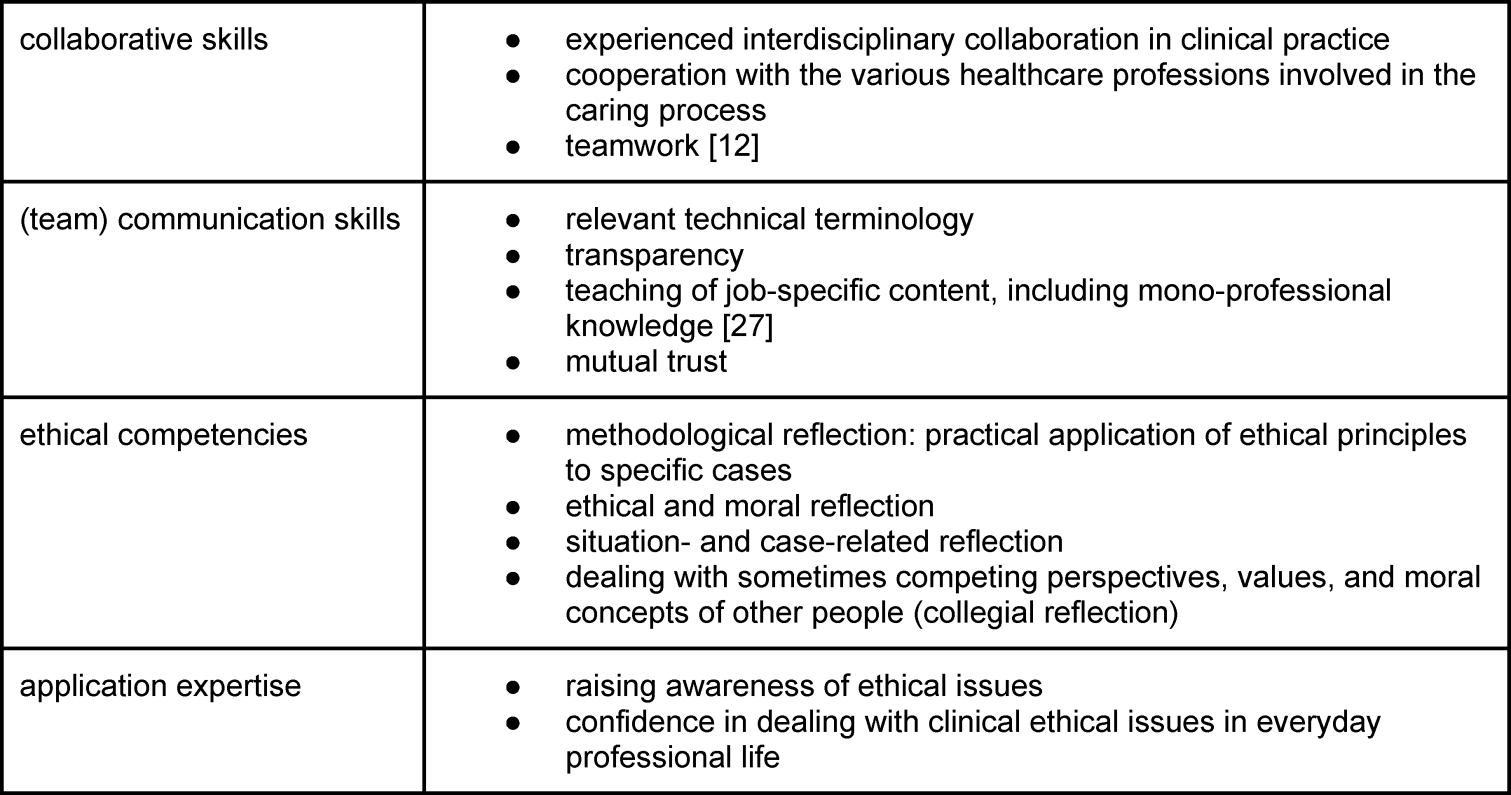
Competencies in (inter)professional identity formation

**Table 2:**
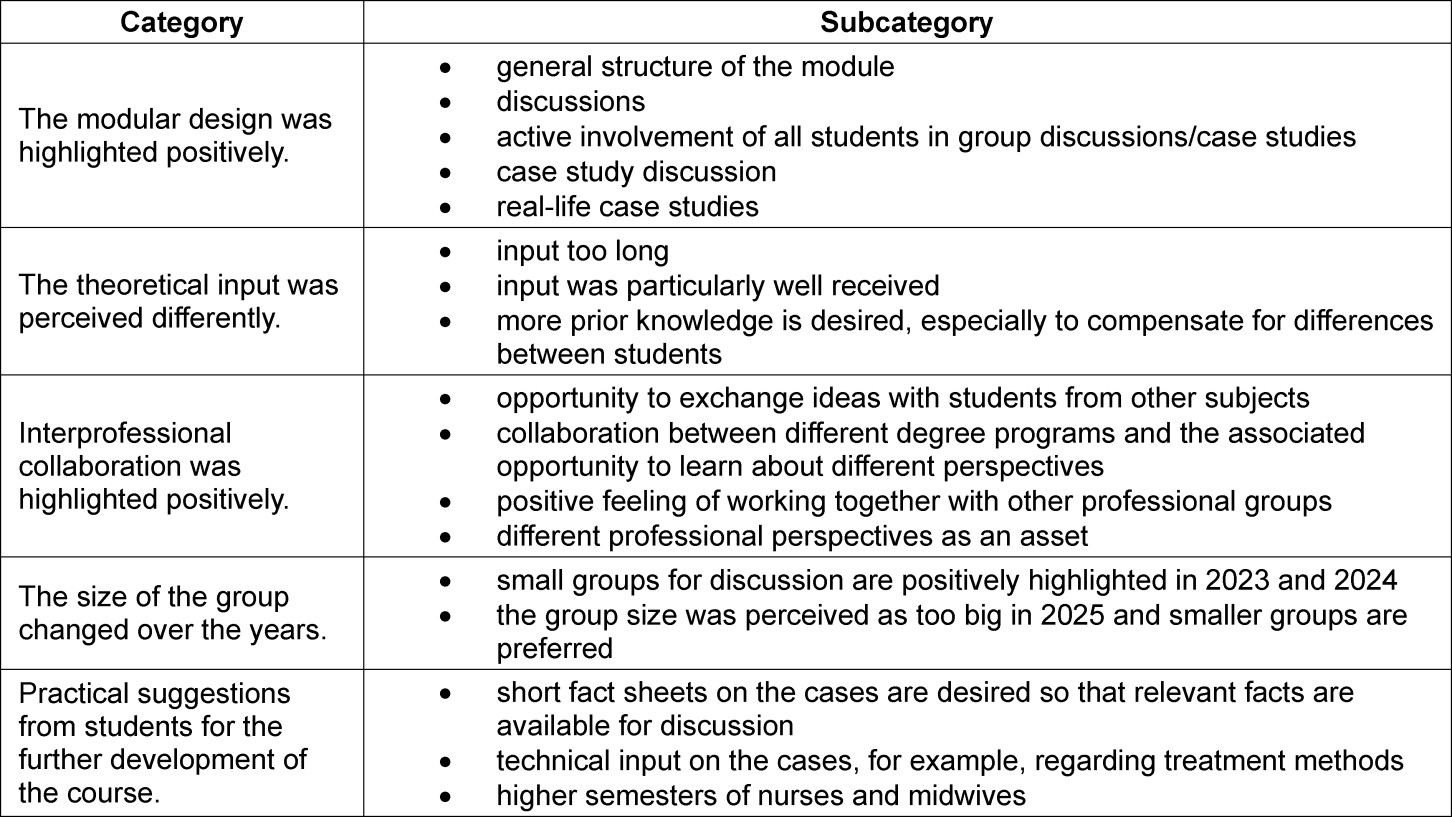
Selected qualitative results from the open-ended questions
